# Quantifying the global contribution of alcohol consumption to cardiomyopathy

**DOI:** 10.1186/s12963-017-0137-1

**Published:** 2017-05-25

**Authors:** Jakob Manthey, Sameer Imtiaz, Maria Neufeld, Margaret Rylett, Jürgen Rehm

**Affiliations:** 1 Institute of Clinical Psychology and Psychotherapy, Technische Universität Dresden, Chemnitzer Str. 46, 01187 Dresden, Germany; 2 Institute for Mental Health Policy Research, CAMH, 33 Russell Street, Toronto, ON M5S 2S1 Canada; 3 Campbell Family Mental Health Research Institute, 250 College Street, Toronto, ON M5T 1R8 Canada; 4 Institute of Medical Science (IMS), University of Toronto, Faculty of Medicine, Medical Sciences Building, 1 King’s College Circle, Room 2374, Toronto, ON M5S 1A8 Canada; 5 Department of Psychiatry, University of Toronto, 250 College Street, 8th floor, Toronto, ON M5T 1R8 Canada; 6 Dalla Lana School of Public Health, University of Toronto, 155 College Street, 6th floor, Toronto, ON M5T 3M7 Canada; 7 PAHO/WHO Collaborating Centre for Mental Health and Addiction, 33 Russell Street, Toronto, ON M5S 2S1 Canada

**Keywords:** Cardiomyopathy, Population-attributable fraction, Mortality, Global burden of disease, Alcohol, Alcohol use disorder, Comparative risk assessment

## Abstract

**Background:**

The global impact of alcohol consumption on deaths due to cardiomyopathy (CM) has not been quantified to date, even though CM contains a subcategory for alcoholic CM with an effect of heavy drinking over time as the postulated underlying causal mechanism. In this feasibility study, a model to estimate the alcohol-attributable fraction (AAF) of CM deaths based on alcohol exposure measures is proposed.

**Methods:**

A two-step model was developed based on aggregate-level data from 95 countries, including the most populous (data from 2013 or last available year). First, the crude mortality rate of alcoholic CM per 1,000,000 adults was predicted using a negative binomial regression based on prevalence of alcohol use disorders (AUD) and adult alcohol *per capita* consumption (APC) (*n* = 52 countries). Second, the proportion of alcoholic CM among all CM deaths (i.e., AAF) was predicted using a fractional response probit regression with alcoholic CM crude mortality rate (from Step 1), AUD prevalence, APC per drinker, and Global Burden of Disease region as predictions. Additional models repeated these steps by sex and for the wider Global Burden of Disease study definition of CM.

**Results:**

There were strong correlations (>0.9) between the crude mortality rate of alcoholic CM and the AAFs, supporting the modeling strategy. In the first step, the population-weighted mean crude mortality rate was estimated at 8.4 alcoholic CM deaths per 1,000,000 (95% CI: 7.4–9.3). In the second step, the global AAFs were estimated at 6.9% (95% CI: 5.4–8.4%). Sex-specific figures suggested a lower AAF among females (2.9%, 95% CI: 2.3–3.4%) as compared to males (8.9%, 95% CI: 7.0–10.7%). Larger deviations between observed and predicted AAFs were found in Eastern Europe and Central Asia.

**Conclusions:**

The model proposed promises to fill the gap to include AAFs for CM into comparative risk assessments in the future. These predictions likely will be underestimates because of the stigma involved in all fully alcohol-attributable conditions and subsequent problems in coding of alcoholic CM deaths.

**Electronic supplementary material:**

The online version of this article (doi:10.1186/s12963-017-0137-1) contains supplementary material, which is available to authorized users.

## Background

Alcohol consumption is a major risk factor for the global burden of disease [1–3]. It has causal relationships with more than 200 three-digit International Statistical Classification of Diseases and Related Health Problems 10^th^ Revision (ICD-10; [4]) disease and injury categories [3]. Two related dimensions of alcohol consumption have been shown to impact disease and injury: (average) level of alcohol consumption and heavy drinking occasions [5–7]. Among the causally related disease and injury categories, there are about 40 that are fully (i.e., 100%) alcohol-attributable, such as alcohol use disorders (AUD; for a list of all fully alcohol-attributable disease and injury categories see [6]). However, many of the fully alcohol-attributable disease and injury categories have not been included in the Global Burden of Disease estimates to date, as there are no global data for smaller causes of death [8], given the unavailability of vital registries for the majority of the global population [9].

To date, the contribution of alcohol to cardiomyopathy (CM) has not been quantified globally. CM (ICD-10 I42) denotes a disease of the heart muscle, reducing its ability to pump blood to the rest of the body. There are multiple forms of CM with different etiologies, but chronic, heavy alcohol consumption is associated with dilated CM, as ethanol acts as a toxin to weaken the heart muscle [10–15]. There is sufficient evidence for causality, based on experimental evidence of the toxic effect of alcohol on muscles and cardiac indicators [16, 17]; and there is even a category of alcoholic cardiomyopathy (ACM) in ICD-10 (I42.6) [4]. ACM has been known since the mid-19th century (e.g., [18]; for an overview of historical accounts see [19]), as detailed by a Munich pathologist, who labeled the phenomenon the “Münchner Bierherz” (the Munich beer heart), a disease characterized by cardiac dilatation and hypertrophy due to heavy consumption of beer over time [20]. However, for the reasons indicated above, the category of ACM is not part of global statistics.

To quantify the relationship between alcohol consumption and CM for future comparative risk assessments, the usual procedure would be to conduct a meta-analysis on the dose-response relationship between level of alcohol consumption and risk of CM, as has been done for all the other disease and injury categories partially attributable to alcohol [6, 7]. In addition, as cardiovascular outcomes are often impacted by patterns of drinking (e.g., [21]), the potential impact of this dimension would be estimated as well. While a recent systematic review did not find enough empirical studies to quantify either relationship [22], results from the identified studies suggested a threshold relationship between alcohol use and CM with a potential dose-response relationship for different levels of heavy consumption. With regard to specific drinking characteristics, heavy drinking (defined as > 80 g pure alcohol/day) over a decade or more [23] was repeatedly found as key risk factor for developing CM (see also [22, 24, 25]). Based on these findings, predicting ACM and/or modeling the relationship between ACM and CM using aggregate alcohol and mortality data was expected to be feasible.

While a population measure of alcohol consumption such as the rate of consuming more than 80 g pure alcohol on average per day over time would be best used to model ACM, this indicator is not available globally. Instead, prevalence of AUD and heavy episodic drinking, as well as adult alcohol *per capita* consumption (APC) can be considered potential substitute measures to predict the alcohol-attributable fraction (AAF) for CM. In fact, APC is closely linked to heavy drinking, as the distribution of drinking follows a gamma distribution and its mean determines the spread (in a one-parameter distribution). Thus, APC directly corresponds to the proportion of heavy drinkers [26, 27].

Predictions for both CM and the larger category of CM in the Global Burden of Disease (GBD) study [28] were performed in this feasibility study. However, CM is responsible for 84% of deaths in the larger GBD category of CM based on the countries included in the present study, and thus the AAFs were not expected to differ widely.

## Methods

We followed the Guidelines for Accurate and Transparent Health Estimates Reporting in the presentation of the global health estimates [29] (detailed checklist can be found in the Additional file 1).

### Data sources

We sought to establish a model for estimating AAF for CM based on data from 48 of the 50 most populous countries (exceptions: Sudan and Myanmar), as well as from all countries of the World Health Organization (WHO) European Region and select countries from other WHO regions (total of 95 countries included). The selection of countries was guided by three considerations: 1) we wanted to ensure that the methodology could be used for global estimates, where 48 of the 50 most populous countries included 86% of the global population in 2015; 2) we wanted to include Eastern European countries, where AAF for CM can be high (e.g., 67% found in a city of Russia [30]; see also Table 3 below); and 3) we wanted to ensure global spread. Altogether the selected countries represented 91% of the global population 15 years and older in 2015.

Alcohol exposure data (heavy episodic drinking, AUD, APC) were taken from the Global Information System on Alcohol and Health of WHO [31]; for the percentage of pure alcohol consumed by men and women, we relied on data from the Global Status Report on Alcohol and Health [2]. Mortality data from 2013 (or last available year) were taken from the WHO Mortality Database [32], with the exception of Russia [33], Slovenia [34], and Ukraine [personal communication of Dr. Andriy Samokhvalov]. Additionally, we used UN data for population size [35], as well as World Bank Gross Domestic Product (GDP) Purchasing Power Parity (PPP) corrected per capita data for measuring comparative wealth of countries [36]. Countries were classified into GBD regions, as per the methodology of the Institute for Health Metrics and Evaluation [37]. All data were matched to year of mortality data (63.3% = 2013) for each country and referred to the adult population (15 years or older). All data were obtained from publicly available international databases that have been previously used in the modeling of Global Health Estimates [38] or within the Global Burden of Disease studies (e.g., [39]). We did not expect any systematic bias in the data used for this study, except for the underestimation of ACM because of stigma and coding problems (discussed in detail below).

### Modeling strategy

The AAFs for CM were modeled in a two-step procedure using aggregate data from 95 countries. In this procedure, we modeled the crude mortality rate for ACM per year (i.e., number of ACM deaths per 1,000,000 adults per year, where adults were defined as 15 years and older to correspond to the APC definition), which was subsequently used in the second step to predict the proportion of ACM deaths among all CM deaths (i.e., the alcohol-attributable fraction – AAF). The rationale for this two-step procedure was based on high correlations between crude mortality rate and the AAFs. Results from Spearman correlations between these right-skewed variables (*N* = 49; *p* = 0.93, 95% confidence interval (CI): 0.88–0.96; for women: *p* = 0.98, 95% CI: 0.97–0.99; for men: *p* = 0.93, 95% CI: 0.88–0.96) supported the reasoning of this two-step strategy.

Prior to model building, bivariate Spearman correlations between potential predictors and both outcomes were examined (see results in Table 1, see scatterplots in Additional file 2). Correlations between the predictors and with crude mortality rate ranged between *p* = 0.36 and *p* = 0.69. Further, large correlations were observed between APC total, prevalence of AUD, and heavy episodic drinking (0.71 < *p* < 0.76).

**Table 1:** Spearman correlation matrix of ACM crude mortality rate, AAFs of CM deaths, and potential predictors

	APC total	APC among drinkers	AUD prevalence	HED prevalence	ACM crude mortality rate	AAFs of CM deaths
APC total	1	/	/	/	/	/
APC among drinkers	0.36	1	/	/	/	/
AUD prevalence	0.71	0.09	1	/	/	/
HED prevalence	0.76	0.00	0.74	1	/	/
ACM crude mortality rate	0.69	0.49	0.41	0.40	1	/
AAFs of CM deaths	0.52	0.37	0.38	0.36	0.93	1

*APC* alcohol per capita, *AUD* alcohol use disorder, *HED* heavy episodic drinking, *ACM* alcoholic cardiomyopathy, *AAFs* alcohol-attributable fractions, *CM* cardiomyopathy

Bivariate correlations based on all available data for each pair

In the first step, the country-specific ACM crude mortality rate was modeled using negative binomial regression. The prediction initially included prevalence of AUD and heavy episodic drinking, as well as total APC. However, heavy episodic drinking was removed from the final model (see eq. 2 below), as it did not improve its predictive accuracy over the simpler model (see below for a description of assessment of goodness of fit; difference in Pseudo-R^2^ = 0.002; likelihood ratio test: Chi^2^(1) = 0.55, *p* = 0.46). The results of the first-step model were integrated into one variable combining the predicted (*N* = 43 countries) and observed (*N* = 52 countries) crude mortality rates for ACM. In the second step, the AAFs were predicted using the crude mortality rate for ACM from Step 1, as well as prevalence of AUD and heavy episodic drinking, APC per drinker, and GBD region using a fractional response probit regression [40]. As in the first-step model, we excluded heavy episodic drinking from the final model (see eq. 3 below) because accuracy of predictions could not be improved by retaining it (difference in Pseudo-R^2^ = 0.002; likelihood ratio test not meaningful for robust standard error models). We additionally tested the contribution of economic wealth by the inclusion of GDP PPP into the model, but it did not improve the accuracy of the prediction.

Eq. 1: negative binomial regression written as generalized linear model

1 $$ \mathrm{A}\mathrm{C}\mathrm{M}\ \mathrm{crude}\ \mathrm{mortality}\ \mathrm{rate}= f\left({\beta}_0+{\beta}_1 \ast AUD\  prevalence+{\beta}_2\ast APC\  total\right) $$

*Link function*: log

*Distribution*: negative binomial

*Variance*: *Var* [*Y*|*x*] = *μ* + *αμ* ^*2*^

Eq. 2: fractional response probit regression written as generalized linear model

2 $$ \mathrm{P}\left(\mathrm{AAF}=1\right)= f\left({\beta}_0+{\beta}_1* crude\kern0.5em  mortality\kern0.5em  rate+{\beta}_2*\kern0.5em  AUD\kern0.5em  prevalence+{\beta}_3* APC\kern0.5em  drinker+{\beta}_4* GBD\kern0.5em  region\right) $$

*Link function*: probit

*Distribution*: binomial

In addition to this model (Model 1), the same analyses were performed separately by sex (Model 2), as there is evidence for a higher risk of ACM for women compared to men given the same level of drinking (for sex differences see [41] and [42]). However, sex-specific ACM crude mortality rates could not be obtained for Ukraine, reducing the available observations for these rates in the first step to 51 countries. In two further models (Models 3 and 4), the second step was repeated with the same predictors for the AAFs using the broader GBD definition of CM [38]. However, some of the ICD-10 categories that were part of the broader GDB definition of CM were not included, as they were not available from the WHO Mortality Database. Similar to the first two models, this was performed for the entire population (Model 3) and separately by sex (Model 4).

To assess the goodness of fit for all models, we used the pseudo R^2^ methods (likelihood ratio of the full model compared to the null model) [43]. In addition, we compared predicted and observed indicators for both steps. An absolute difference of at least 10 deaths per 1,000,000 (Step 1) and of 5% in the proportion of ACM deaths among all CM deaths (Step 2) were considered relevant deviations. Systematic differences in GDP PPP (using *t*-test), alcohol use disorder prevalence (using *t*-test), and in the regional distributions (using standardized deviations larger than 1.96) were identified and described between countries within and beyond these thresholds.

In order to generalize the estimates, outcomes from both steps (crude mortality rate and AAFs), were weighted by the population size of the given country. The weighted variance was obtained using the standard formula for sums of weighted variances (see eq. 3 below), assuming that the covariance between countries was zero. Variances were then used to estimate standard error and 95% confidence intervals. Altogether, the rates (Step 1) and proportions (Step 2) were presented in three ways: 1) unweighted predicted rates/proportions; 2) predicted rates/proportions weighted for population size; and 3) observed and predicted (including observed values first and if missing – predicted values) rates/proportions weighted for population size.

Eq. 3: Estimation of population weighted variance

3 $$ \mathrm{V}\mathrm{a}\mathrm{r}\left({\displaystyle \sum_i^n}{a}_i{X}_i\right) = {\displaystyle \sum_{i=1}^n}{a}_i^2\  V a r\left({X}_i\right)+2{\displaystyle \sum_{1\le i}}{\displaystyle \sum_{< j\le n}}{a}_i{a}_j C o v\left({X}_i,{X}_j\right) $$

Analyses were performed using Stata 14.0 [44] and R [45]. The file and the corresponding syntax for all calculations can be found in the Additional files 3 and 4.

## Results

A two-step model predicted (1) the ACM crude mortality rate and (2) the AAF for CM deaths in 95 selected countries.

### Step 1: Crude mortality rate of ACM

In Model 1 - Step 1, the ACM crude mortality rate was predicted based on 52 countries using a negative binomial regression (see eq. 1). In Table 2, both observed (*N* = 52) and predicted (*N* = 95) crude mortality rates are presented, while all model parameters (intercept, coefficient, dispersion parameter, Pseudo R^2^) can be found in the appended (Additional file 5) Web Table (Sheet “Model 1 (total)”). The predicted rates ranged between 0.7 (Saudi Arabia) and 267.9 (Belarus) ACM deaths per 1,000,000. Comparing the observed and predicted rates, the mean absolute difference amounted to 16.8 ACM deaths per 1,000,000 (min: 0.4, max: 150.2, median: 7.3), with 37 countries (71.2%; representing 81.8% of the entire population with observed ACM mortality data) within a range of +/- 10 ACM deaths per 1,000,000 adults (countries beyond the threshold are highlighted in Table 2). Comparing countries within and beyond this threshold, we found that countries with large deviations between observed and predicted ACM crude mortality rates had a similar GDP PPP ($30,542 versus $35,820, *t*-test: *p* = 0.382) but higher AUD prevalence (4.9% versus 9.0%, *t*-test: *p* = <0.001). With respect to deviations by region, higher deviations were more common in Eastern European countries (one country within threshold versus five countries beyond threshold; standardized deviation = 3.13). Excluding Eastern European countries, the mean absolute difference between observed and predicted crude mortality rates fell from 16.8 to 11.5 cases per 1,000,000 (min: 0.4, max: 149, median: 5.6).

**Table 2:**
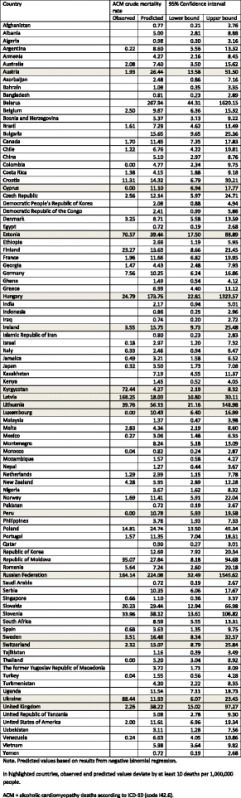
Observed and predicted ACM deaths per 1,000,000 people, by country

For the included countries, an unweighted average of 15.2 ACM deaths per 1,000,000 was predicted (95% CI: 9.2–21.2). The weighted ACM crude mortality rate based on predicted values only was estimated to be 11.4 per 1,000,000 (95% CI: 10.1–12.6), whereas the mean weighted ACM crude mortality rate combining both observed and predicted values was estimated to be 8.4 ACM deaths per 1,000,000 (95% CI: 7.4–9.3). The results of sex-specific predictions (Model 2, Step 1) can be found in the appended (Additional file 5) Web Table (Sheets “Model 2 (female)” and “Model 2 (male)”). The weighted average ACM mortality rate of observed and predicted deaths was estimated to be 2.8 (95% CI: 2.4–3.3) and 12.9 (95% CI: 11.3–14.6) deaths per 1,000,000 for females and males, respectively. Both the observed and predicted crude mortality rates in males were greater than or equal to those of females in all included countries.

### Step 2: Proportion of ACM deaths among all CM deaths

The proportion of ACM deaths among all CM deaths was predicted in Step 2 of Model 1. In addition to all observed proportions (*N* = 50), Table 3 contains all predicted (*N* = 95) proportions, which ranged between 0.0% (Indonesia) and 86.0% (Belarus). Comparing the observed and predicted proportions, the mean absolute difference amounted to 4.7% (min: 0.0%, max: 30.0%, median: 1.9%), with 39 countries (78%; representing 95.8% of entire population with observed AAF data) within a +/- 5% range (countries beyond the threshold are highlighted in Table 3). Comparing countries within and beyond this threshold, we found that countries with large deviations between observed and predicted AAF had a similar GDP PPP ($34,811 versus $25,217; *t*-test: *p* = 0.153) and comparable AUD prevalence (5.7% versus 6.4%; *t*-test: *p* = 0.509). However, Eastern European (one country within threshold versus three countries beyond threshold; standardized deviation = 2.67) and Central Asian (zero countries within threshold versus two countries beyond threshold; standardized deviation = 2.72) countries were overrepresented in those countries beyond the threshold.

**Table 3:**
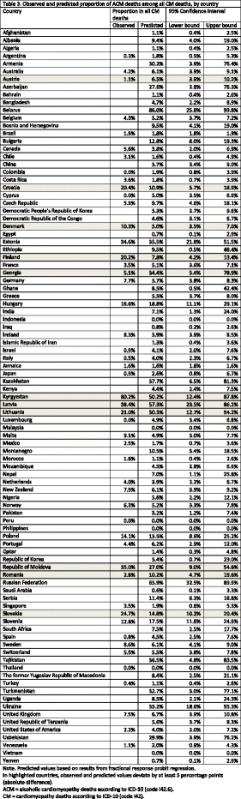
Observed and predicted proportion of ACM deaths among all CM deaths, by country

For the included countries, an unweighted mean AAF of 10.8% (95% CI: 10.3–11.4%) was predicted. The population weighted AAF based on predicted values only was estimated to be 7.1% (95% CI: 5.5–8.6%) of all CM deaths. Applying population weights to the combination of observed and predicted values, the mean AAF was calculated to be 6.9% (95% CI: 5.4–8.4%).

The results of sex-specific predictions (Model 2, Step 2) can be found in the appended Web Table (Sheets “Model 2 (female)” and “Model 2 (male)”). The weighted average AAF of observed and predicted proportions were estimated to be 2.9% (95% CI: 2.3–3.4%) and 8.9% (95% CI: 7.0–10.7%) for females and males, respectively.

The appended (Additional file 5) Web Table also presents results from Models 3 and 4, which incorporate the broader GBD definition of CM. Based on these models, the weighted average AAF combining observed and predicted proportions of ACM deaths among all CM deaths was estimated to be 5.9% (95% CI: 4.5–7.2%) for the entire population, 2.6% (95% CI: 2.0–3.1%) for females, and 7.9% (95% CI: 6.2–9.6%) for males.

## Discussion

We were able to estimate the AAF for CM, as well as for the GBD definition of CM. Altogether, the models fit reasonably well. However, we have identified several limitations.

In predicting the ACM crude mortality rate, marked deviations from observed values were detected for countries with high alcohol use disorder prevalence and for some countries from Eastern Europe. In these countries, the observed ACM mortality rates varied more than in any other region. Take, for example, the three Baltic countries, Latvia, Estonia, and Lithuania. Here, variations in AUD prevalence (7.7–10.2%) and APC (12.0–17.9) clearly did not correspond to the variations in the observed ACM crude mortality rate (39.8–168.3). We acknowledge that other factors may be necessary for a more accurate modeling of ACM mortality in this region (see below for potential factors). However, for the remaining countries, we found the predictions to fit sufficiently.

In modeling the primary outcome (AAFs), accurate predictions were yielded for the majority of countries. However, similar to the first step, inaccurate estimates were pronounced among Eastern European countries in addition to Central Asian countries. Again, a large variation in observed AAFs in these countries could not be sufficiently accounted for by the included covariates. While the observed and predicted AAFs were highest in these countries (e.g., Kyrgyzstan: 80%, Moldova: 55%), they represent a relatively small share of the global population. Therefore, the proposed quantification was accurate (deviation of observed and predicted AAF < = 5%) for 95.8% of the examined population.

In order to understand the limitations of the model, one should have a closer look at the data sources. The first major limitation is related to ACM mortality data, which may be subject to coding errors. As persons with ACM are likely to have other end-organ damages, attribution of a person’s death to a single disease category without any autopsy can be quite challenging [46]; and then there is the attribution to alcohol as well (see next paragraph). In some former Soviet countries like Belarus [47], Kyrgyzstan [48], Russia [49, 50], and Ukraine [51], autopsies are obligatory for many deceased people. While autopsies can determine CM as the cause of death, additional information is required on heavy drinking in order to identify ACM, e.g., via official AUD registries. However, in Russia, alcohol diagnoses either have to be established by an addiction medicine physician or require a documented history of alcohol use problems with the patient being formerly registered in the state-run addiction treatment system [52]. Specifically, for the diagnosis of ACM, chronic alcoholism is one of the required criteria, which is why a substantial fraction of ACM deaths might be falsely coded as nonspecific cardiomyopathies [53], as the physician conducting the autopsy may not always check for this information. As only two out of 10 people with AUD seek treatment worldwide [54], this can be a source of bias in ACM mortality data, despite routinely performed autopsies.

Further and not restricted to ACM, there is a general problem with ICD-10 categories fully attributable to alcohol, i.e., those categories with “alcohol” or “alcoholic” in their names [4]. There is a high stigma attached to alcohol use disorders [55], even compared to other mental disorders [56]. The specific stigma against alcohol use disorders seems to have persisted over the past decades [57], even though medical treatment seems to be more acceptable [58] and more people endorse a neuroscientific view of mental disorders, including alcohol use disorders [59, 60]. As a consequence of this rather universal stigma in our societies [55], fully alcohol-attributable disease categories are likely to be underreported, as a number of studies have demonstrated. Most prominently, in a study in 12 cities in 10 countries, Puffer and Griffith [61] found that after triangulating data on death certificates with data from hospital records and interviews of attending physicians or family members, the number of deaths with alcoholic liver cirrhosis more than doubled, with the majority of new cases being detected under categories of cirrhosis that do not mention alcohol. This underreporting of alcoholic liver cirrhosis has persisted in later studies as well [46, 62–64], and it seems to be the case for all disease categories fully attributable to alcohol use [46, 65]. More specifically for ACM, one study estimated the amount of underestimation to be about 30% [64].

As such, ACM mortality rates may have been underestimated due to stigma and coding problems. Despite these issues and the limited quality of WHO mortality data from some countries [9, 66], we argue that using these data is still the best possible approach to obtain estimates of AAFs for CM mortality. Trivially, we expect precision of our model to improve with a growing number of countries with vital registries and an increasing accuracy of reported mortality data (e.g., using autopsies to validate death certificates, see [67]). However, autopsies alone will only be able to determine the alcoholic part or ACM in very few cases, and stigmatization of 100% alcohol-attributable diseases will remain a problem. Thus, it may be valuable to find and include stigmatization indicators in the model in the future to correct for stigmatization-attributable underestimations.

The second major limitation of our model is related to the degree of uncertainty inherited in aggregated alcohol measures. Accurate measurements of country-specific AUD rates are mostly lacking and have to be estimated instead. Moreover, these measurements are assessing a stigmatized disease through self-reports based on symptoms, which are culturally specific, thus introducing considerable bias in existing data [68, 69]. Total APC as an indicator has less bias, as it is mostly composed of sales, production, export, and import data, but the unrecorded component also may introduce significant bias [70]. In theory, the prediction model could be improved by using estimates for very heavy drinking over an extended period over time, but such data would need to be based on alcohol exposure of representative cohorts over decades, which simply do not exist.

## Conclusion

Based on the crude mortality rate for ACM, alcohol use disorder prevalence and APC per drinker, estimation of AAFs for CM has been shown to be feasible through statistical modeling. However, limitations in data reliability and the limited knowledge of the relationship between alcohol and CM indicate that the proposed modeling strategy is only a first step toward a more comprehensive quantification of the global burden of ACM. We should strive to establish AAFs based on exposure and relative risk [26, 71, 72], similar to the way it has been established for liver cirrhosis [73] to avoid underestimation [74]. However, this is a long-term solution, which will require cohort or case-control studies. In the meantime, we propose to apply the current methodology in order to determine the effects of alcohol consumption on CM in countries without observed AAFs, even if it likely will be an underestimation.

## Additional files

Additional file 1: ACM Quantification_GATHER checklist. (DOCX 17 kb)

Additional file 2: Web figures presenting scatterplots and regression lines of outcomes and predictors. (DOCX 58 kb)

Additional file 3: Stata file. (DTA 89 kb)

Additional file 4: Syntaxes. (ZIP 8 kb)

Additional file 5: Results from all four models including model data. (XLSX 365 kb)

## Abbreviations

AAF: Alcohol-attributable fraction
ACM: Alcoholic cardiomyopathy
APC: (adult) Alcohol *per capita* consumption
CI: Confidence interval
CM: Cardiomyopathy
GBD: Global Burden of Disease
GDP: Gross domestic product
ICD-10: International statistical classification of diseases and related health problems, 10th revision
WHO: World Health Organization
