# Supplementary material for: Quantifying the global contribution of alcohol consumption to cardiomyopathy
Source: Popul Health Metr. 2017 May 25;15:20. doi: 10.1186/s12963-017-0137-1 (PMC5445448; doi:10.1186/s12963-017-0137-1)
Supplement: Supplementary file 1 — ACM Quantification_GATHER checklist. (DOCX 17 kb) [file 12963_2017_137_MOESM1_ESM.docx]

| **GATHER statement for manuscript “Quantifying the global contribution of alcohol consumption to cardiomyopathy”** | | |
| --- | --- | --- |
|  |  |  |
| **Checklist item** | **Reported on page** | **Additional note** |
| **Objectives and funding** |  |  |
| **1** Define the indicator(s), populations (including age, sex, and geographic entities), and time period(s) for which estimates were made. | **6-7** | Under heading “Data sources” |
| **2** List the funding sources for the work. | **14** | Under heading “Funding” |
| **Data Inputs** | | |
| *For all data inputs from multiple sources that are synthesized as part of the study:* | | |
| **3** Describe how the data were identified and how the data were accessed. | **6-7** | Under heading “Data sources” |
| **4** Specify the inclusion and exclusion criteria. Identify all ad‐hoc exclusions. | **6-7** | Under heading “Data sources” |
| **5** Provide information on all included data sources and their main characteristics. For each data source used, report reference information or contact name/institution, population represented, data collection method, year(s) of data collection, sex and age range, diagnostic criteria or measurement method, and sample size, as relevant. | **6-7** | Under heading “Data sources” |
| **6** Identify and describe any categories of input data that have potentially important biases (e.g., based on characteristics listed in item 5). | **6-7; 14-16** | Mentioned in “Data sources,” discussed thoroughly in “Discussion” |
| *For data inputs that contribute to the analysis but were not synthesized as part of the study:* | | |
| **7** Describe and give sources for any other data inputs. | **6-7** | Under heading “Data sources” |
| *For all data inputs:* | | |
| **8** Provide all data inputs in a file format from which data can be efficiently extracted (e.g., a spreadsheet rather than a PDF), including all relevant meta‐data listed in item 5. For any data inputs that cannot be shared because of ethical or legal reasons, such as third‐party ownership, provide a contact name or the name of the institution that retains the right to the data. | **Web Appendix** | All data can be found in the Web Table (Excel file) and prepared as Stata dataset. |
| **Data analysis** | | |
| **9** Provide a conceptual overview of the data analysis method. A diagram may be helpful. | **7-10** | Under heading “Modeling strategy” |
| **10** Provide a detailed description of all steps of the analysis, including mathematical formulae. This description should cover, as relevant, data cleaning, data pre‐processing, data adjustments and weighting of data sources, and mathematical or statistical model(s). | **8-10** | Under heading “Modeling strategy” |
| **11** Describe how candidate models were evaluated and how the final model(s) were selected. | **7-9** (until equation 1) | Under heading “Modeling strategy” and Table 1 |
| **12** Provide the results of an evaluation of model performance, if done, as well as the results of any relevant sensitivity analysis. | **11-13** | Under heading “Step 1: Crude mortality rate of ACM” and “Step 2: Proportion of ACM deaths among all CM deaths” |
| **13** Describe methods for calculating uncertainty of the estimates. State which sources of uncertainty were, and were not, accounted for in the uncertainty analysis. | **10** (equation 3) | Under heading “Statistical analysis” |
| **14** State how analytic or statistical source code used to generate estimates can be accessed. | **Web Appendix** | All codes can be found in the Web Appendix |
| **Results and Discussion** | | |
| **15** Provide published estimates in a file format from which data can be efficiently extracted. | **Web Appendix** | All results are presented in the Web Table |
| **16** Report a quantitative measure of the uncertainty of the estimates (e.g. uncertainty intervals). | **11-13, Table 2 and 3** | 95% confidence intervals are reported for each estimate |
| **17** Interpret results in light of existing evidence. If updating a previous set of estimates, describe the reasons for changes in estimates. | **13-16** | Discussion |
| **18** Discuss limitations of the estimates. Include a discussion of any modelling assumptions or data limitations that affect interpretation of the estimates. | **14-16** | Discussion |
