# Supplementary material for: Quantifying the global contribution of alcohol consumption to cardiomyopathy
Source: Popul Health Metr. 2017 May 25;15:20. doi: 10.1186/s12963-017-0137-1 (PMC5445448; doi:10.1186/s12963-017-0137-1)

***Manuscript: ‘Quantifying the global contribution of alcohol consumption to cardiomyopathy’***

**Title: Web figures presenting scatterplots and regression lines of outcomes and predictors**

**Web Figure 1:** Scatterplot and regression line of total alcohol per capita (APC) with crude mortality rate and alcohol attributable fractions


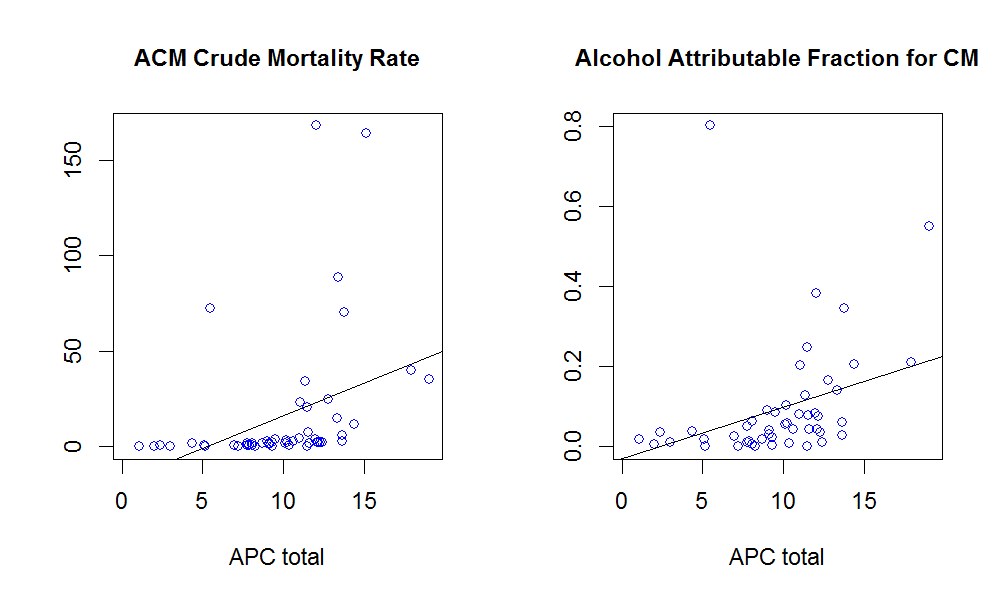


**Web Figure 2:** Scatterplot and regression line of alcohol per capita (APC) per drinker with crude mortality rate and alcohol attributable fractions


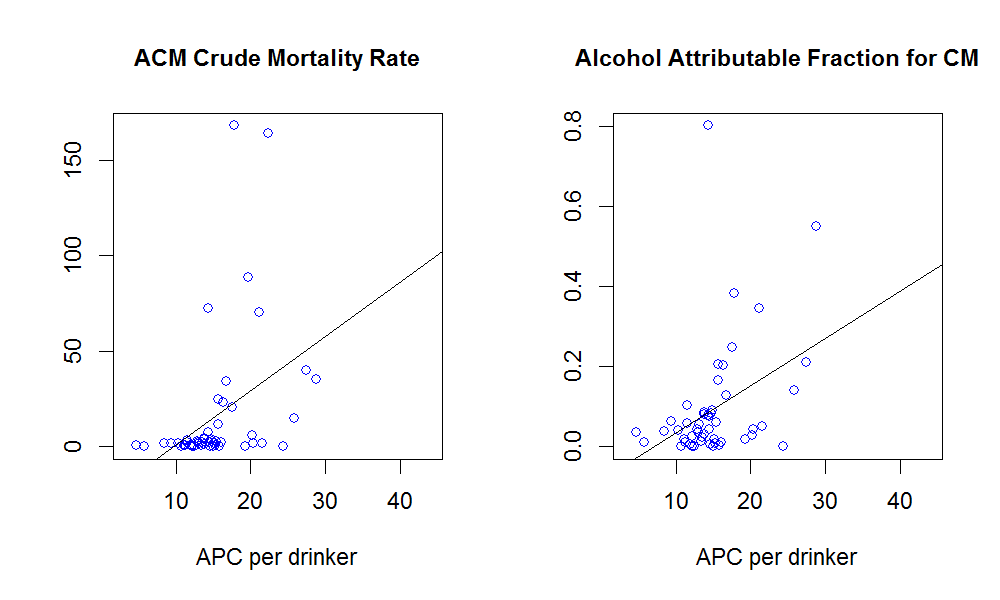


**Web Figure 3:** Scatterplot and regression line of alcohol use disorder prevalence with crude mortality rate and alcohol attributable fractions


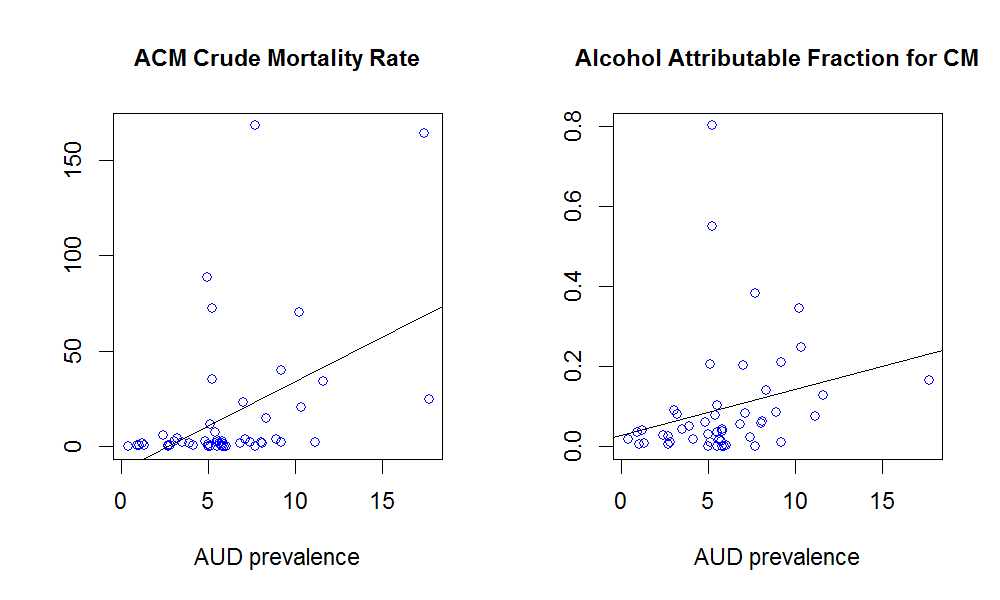


**Web Figure 4:** Scatterplot and regression line of heavy episodic drinking prevalence with crude mortality rate and alcohol attributable fractions


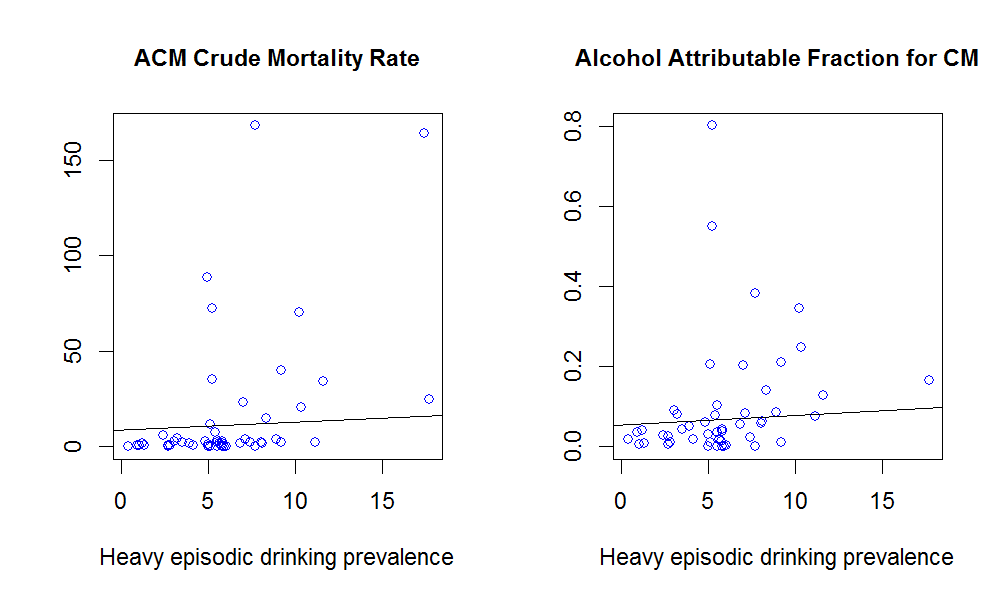

Supplement: Supplementary file 2 — Web figures presenting scatterplots and regression lines of outcomes and predictors. (DOCX 58 kb) [file 12963_2017_137_MOESM2_ESM.docx]
